# The SPIZ study: rationale and protocol of a randomized controlled trial to optimize interdisciplinary care of patients following cellular therapy through a digitally supported, cross-regional and cross-sectoral care model

**DOI:** 10.1007/s00277-026-07290-9

**Published:** 2026-09-26

**Authors:** Martin M. K. Schneider, Gabriele Müller, Sabrina Herbst, Julia Slesaczeck, Roman Schmädig, Franziska Schmidt, Thomas Illmer, Lynn Leppla, Sabine Valenta, Sabina De Geest, Alexandra Teynor, Markus Wolfien, Alice Ernst, Lara Bischof, Jochen Schmitt, Mathias Hänel, Klaus H. Metzeler, Uwe Platzbecker, Vladan Vučinić, Johannes Schetelig, Martin Bornhäuser, Jan Moritz Middeke, Katharina Egger-Heidrich

**Affiliations:** 1 Department of Internal Medicine I, University Hospital Carl Gustav Carus, TUD Dresden University of Technology, Dresden, Germany; 2 Center for Evidence-Based Healthcare, Faculty of Medicine, University Hospital Carl Gustav Carus, TUD Dresden University of Technology, Dresden, Germany; 3 Städtisches Klinikum Dresden, Dresden, Germany; 4 Directorate Nursing, University Hospital Carl Gustav Carus, TUD Dresden University of Technology, Dresden, Germany; 5 Private Practice Hematology and Oncology, Dresden, Germany; 6 Department of Medicine I, Faculty of Medicine, Medical Center - University of Freiburg, University of Freiburg, Freiburg im Breisgau, Germany; 7 Institute of Nursing Science, Department Public Health, Faculty of Medicine, University of Basel, Basel, Switzerland; 8 Nursing Science, Institute for Health Sciences, Faculty of Medicine, University of Tübingen, Tübingen, Germany; 9 Academic Centre for Nursing and Midwifery, Department of Public Health and Primary Care, KU Leuven, Leuven, Belgium; 10 Institute for Agile Software Development, Technical University of Applied Sciences Augsburg, Augsburg, Germany; 11 Institute for Medical Informatics and Biometry, Faculty of Medicine, University Hospital Carl Gustav Carus, TUD Dresden University of Technology, Dresden, Germany; 12 ScaDS.AI Dresden/Leipzig (Center for Scalable Data Analytics and Artificial Intelligence), Dresden/Leipzig, Germany; 13 AOK PLUS, Sternplatz 7, 01067 Dresden, Germany; 14 Department of Hematology and Cellular Therapy, Hemostaseology and Infectious Diseases, University Hospital Leipzig, Leipzig, Germany; 15 Department of Internal Medicine III, Klinikum Chemnitz, Chemnitz, Germany; 16 University Hospital Carl Gustav Carus, TUD Dresden University of Technology, Dresden, Germany; 17 Fraunhofer Institute for Cell Therapy and Immunology IZI, Leipzig, Germany; 18 DKMS Clinical Trials Unit, Dresden, Germany; 19 German Cancer Consortium (DKTK), Partner Site Dresden, Dresden, Germany; 20 German Cancer Research Center (DKFZ), Heidelberg, Germany; 21 National Center for Tumor Diseases Dresden (NCT/UCC), Dresden, Germany; 22 Else Kröner Fresenius Center for Digital Health, TUD Dresden University of Technology, Dresden, Germany; 23 Mildred Scheel Early Career Center, National Center for Tumor Diseases (NCT/UCC) Dresden, Faculty of Medicine and University Hospital Carl Gustav Carus, TUD Dresden University of Technology, Dresden, Germany; 24 University Hospital Carl Gustav Carus Dresden, Fetscherstraße 74, 01307 Dresden, Germany

**Keywords:** Allogeneic hematopoietic cell transplantation, CAR T-cell therapy, post-treatment care, digital health

## Abstract

Allogeneic hematopoietic cell transplantation (alloHCT) and chimeric antigen receptor T (CAR T)-cell therapy represent established therapeutic modalities for selected hematologic malignancies but are associated with substantial morbidity, high healthcare costs, and a considerable risk of severe complications, including death. This necessitates specialized and continuous post-treatment care, which is currently insufficiently standardized and still largely delivered predominantly through in-person and nonintegrated processes. We aimed to develop a comprehensive and innovative post-treatment care program for patients after alloHCT and CAR T-cell therapy based on an interdisciplinary, digitally supported, cross-regional and, cross-sectoral approach, and to evaluate its effectiveness in a randomized controlled trial (RCT). Developed by a multidisciplinary team, the intervention involves case managers and home visits by onco nurses, supported by digital components. These include patient-reported monitoring of vital signs and symptoms via a mobile application, video consultations, and virtual case conferences with community-based hematologists. The evaluation of effectiveness includes both quantitative outcomes, such as mortality and rehospitalization rates, and qualitative data derived from semi-structured interviews. The required ethics approvals were obtained in due time, and the three study centers were successfully initiated in 2024. As of January 2026, recruitment has been completed, and a total of 306 patients were successfully enrolled and randomized in a 1:1 ratio to receive either the SPIZ intervention or routine care. Funded and supported by the Federal Joint Committee (G-BA), a positive evaluation could facilitate the integration of the proposed care model into routine clinical practice, thereby strengthening post-treatment care for patients undergoing cellular therapy across Germany.

**Supplementary Information:**

The online version contains supplementary material available at https://doi.org/10.1007/s00277-026-07290-9.

## Introduction

Cellular therapies like allogeneic hematopoietic cell transplantation (alloHCT) and chimeric antigen receptor T (CAR T)-cell therapy have led to an improvement in prognosis of patients with hematologic diseases [1, 2]. AlloHCT has been established over the past decades as a standard treatment for various hematological diseases [2]. Since 2018, CAR T-cell therapy has been approved for patients with lymphoma and certain types of leukemia in Germany [3, 4]. Both alloHCT and CAR T-cell therapy require a high level of expertise, specific structural requirements such as the accreditation of treatment centers, and substantial personnel resources for the primary hospital stay and post-treatment care [5, 6]. In Germany, regulatory and administrative requirements are governed by a decision of the Federal Joint Committee (Gemeinsamer Bundesausschuss, G-BA) [7].

The continuous increase in the number of patients treated with innovative cellular therapies has led to a growing population requiring post-treatment care [2]. After discharge from the primary hospital stay for cellular therapy, patients undergoing these innovative treatments remain at high risk of mortality and severe complications, including life-threatening infections, organ toxicities, and disease relapse following cellular therapy in general, as well as graft-versus-host disease (GvHD) after alloHCT and cytokine release syndrome (CRS), or neurotoxicity, such as immune effector cell-associated neurotoxicity syndrome (ICANS), after CAR T-cell therapy, in particular. In a large Japanese trial among alloHCT recipients, 2-year non-relapse mortality (NRM) was reported to be 16% [8]. Within a large US-American cohort, 31% of patients required readmission within the first 100 days after alloHCT [9]. Within a German cohort rehospitalizations after alloHCT were as high as 55% [10]. NRM in CAR T-cell therapy was reported to be disease-specific, e.g., reaching 10.6% in mantle cell lymphoma patients [11]. 41% of CAR T-cell therapy patients needed rehospitalization within an US-American cohort with 9% intensive care unit readmissions [12].

Due to their complexity and in view of stringent quality assurance measures, alloHCT and CAR T-cell therapy are performed in certified treatment centers [7]. Post-treatment care is challenged by the necessity for many patients, particularly those residing in rural areas of Germany, to travel significant distances (up to 200 km) to attend regular outpatient appointments or to access the cell therapy center in the event of complications possibly resulting in significant financial and physical stress for patients and their healthcare providers [13]. Furthermore, hours spent in waiting rooms and taxis could be a source of infection in a highly susceptible population.

In a previous study conducted in Boston, Massachusetts, USA, shared post-treatment care between the cell therapy center and community-based physicians after alloHCT was found to be safe, and resulted in non-inferior early NRM and improvement of short-term quality of life (QoL) at day 100; however, no long-term QoL benefit was observed at six months [14]. In Germany, structured post-treatment care programs for patients after cellular therapy currently neither exist nor receive funding from statutory health insurance.

In preparation to design this study protocol, a structured exchange was conducted with patient advocates and cell therapy centers planning to participate in the study. This exchange revealed substantial heterogeneity in post-treatment care across centers and physicians, as well as limited collaboration between healthcare sectors, e.g., hematologists at the cell therapy center, community-based hematologists, and general practitioners.

## Methods

**Fig. 1:**
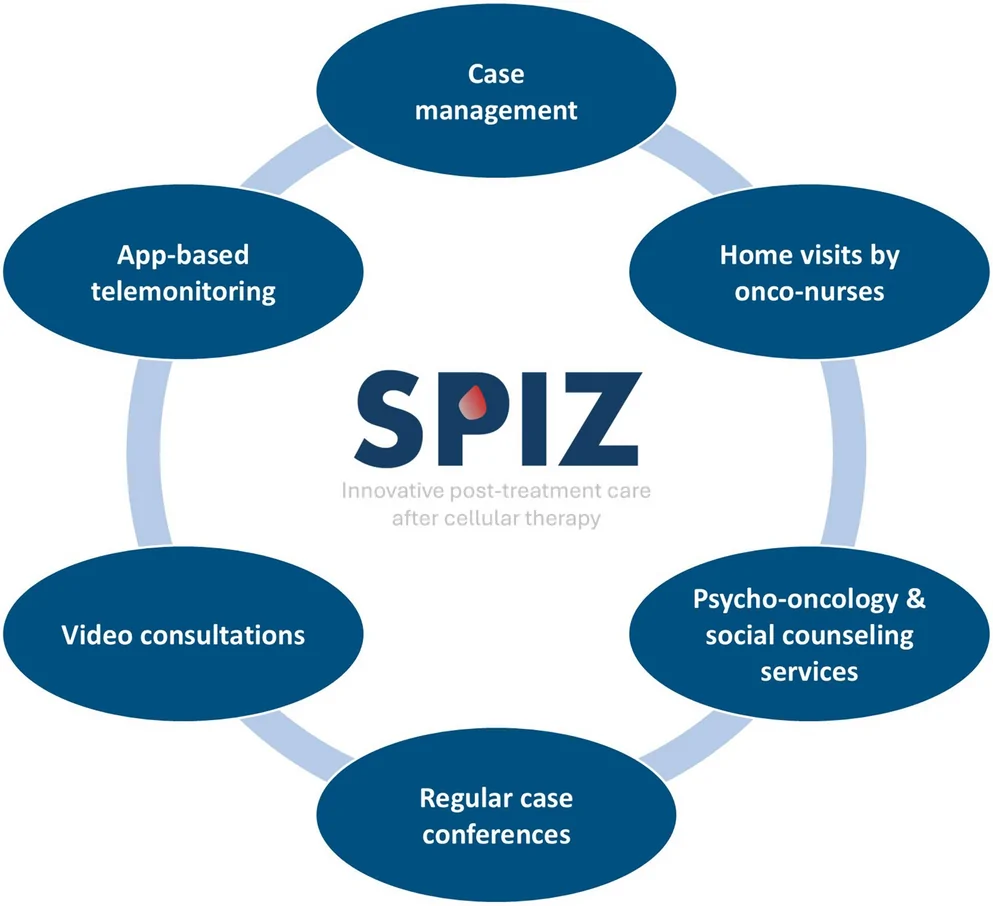
Components of the SPIZ program: By integrating case managers and onco nurses, additional personnel resources are mobilized as part of a specialized care team. Case managers serve as the primary point of contact and as coordinators along the care pathway, whereas onco nurses provide advanced clinical support through home visits and nursing consultations. The psycho-oncology and social counseling services offer psychological support and help to navigate social and legal complexities. App-based telemonitoring via the SMILe system and video consultations constitute digital components of the SPIZ program. Regular case conferences with community-based hematologists facilitate cross-sectoral coordination of care. Onco nurse: oncology nurse; SMILe: SteM-cell-transplantatIon faciLitated by eHealth, SPIZ: cross-sector care for patients with hematological diseases following innovative cell therapy

Based on these challenges, we developed the SPIZ program (cross-sector care for patients with hematological diseases following innovative cell therapy, German: Sektorenübergreifende Versorgung von Patientinnen und Patienten mit hämatologischen Erkrankungen nach innovativer Zelltherapie) as a multidisciplinary collaboration of hematologists, nursing scientists, health services researchers, computer scientists, and patient advocates, comprising the following components (Fig. 1):

1. Case Managers serve as the primary contacts for patients and their families, as well as for community-based hematologists and general practitioners. They organize and coordinate diagnostic and follow-up appointments, facilitate communication across healthcare sectors (e.g., between inpatient and outpatient healthcare providers), and arrange virtual case conferences with community-based hematologists. In addition, case managers provide technical support for the use of the program’s digital components.
2. The SMILe App (SteM-cell-transplantatIon faciLitated by eHealth) enables patients to document vital parameters and relevant clinical symptoms on a daily basis. In addition, patients may use a free-text comment field within the app to request a callback. SMILe Care provides the interface for oncology nurses (onco nurses) to conduct continuous symptom telemonitoring [15].
3. Onco nurses monitor patient-reported data entered in the app using a specialized application (SMILe Care) and present it to the treating hematologist in the event of abnormalities. They conduct regular home visits to assess patients’ health status, collect blood samples, and evaluate the home environment, for example with regard to hygiene standards and the need for supportive care aids. These home visits partially replace routine post-treatment care visits at the hospital.
4. The psycho-oncology service provides specialized psychological support to facilitate patients’ coping with illness and the emotional processing of their experiences. The social counseling service offers professional assistance in addressing personal and social challenges. Contact with both services is typically established during the initial inpatient stay and is maintained to ensure continuous, needs-based support throughout the SPIZ program.
5. Video consultations with the responsible hematologist at the cell therapy center are conducted to complement in-person and home-based care and to ensure continuous specialist involvement. These consultations are typically scheduled following a home visit by an onco nurse but may also be arranged on short notice to provide additional telemedical support whenever clinically indicated.
6. Fortnightly online case conferences are held to discuss patients’ post-treatment care plans within an interdisciplinary, multiprofessional setting by hematologists from the cell therapy centers, community-based hematologists, professionals from psycho-oncology and social counseling services, as well as SPIZ case managers and onco nurses. These conferences aim to ensure coordinated, comprehensive care and to prevent loss of information across care sectors.

Case managers and onco nurses are registered nurses who have completed a three-year professional nursing program and specialist training in oncology or an equivalent qualification. Certification in case management, or a commitment to obtain such certification, is also required for the case management role. Before assuming study-related responsibilities, staff receives project-specific training covering the study procedures, use of the SMILe App and monitoring dashboard, standardized assessments, documentation requirements, and escalation procedures. The average caseload is 25 subjects in the intervention group over the whole study period per full-time-equivalent. Staff works within multidisciplinary teams comprising case managers, onco nurses, physicians, and the project coordinator. Organizational oversight is provided by the project coordinator, while clinical supervision and clinical decision-making remains the responsibility of the hematologists at the respective center.

The effectiveness of SPIZ is currently being evaluated in a randomized controlled trial (RCT) conducted in accordance with study protocol version 5 (dated November 17, 2023): In the intervention group, patients receive the measures described above, while the control group undergoes post-treatment care according to institutional standards. During the early post-cellular therapy period (day 30 to day 100), care is particularly intensive and varies between patients depending on individual needs. After approximately day 100, the intensity of medical and nursing contacts decreases according to the individual clinical trajectory in both the intervention and control arms.

To ensure coverage of a large service area with substantial rural representation, the SPIZ program is implemented at all three cell therapy centers in the federal state of Saxony, Germany: University Hospital Dresden, University Hospital Leipzig, and Klinikum Chemnitz. Structured post-treatment care plans were implemented to standardize and coordinate patient care (supplementary Figs. 1 and 2).

### Eligibility criteria

The target population comprises all adult patients (≥ 18 years) after alloHCT or CAR T-cell therapy.

#### The primary study population are patients who

- have been treated at one of the three cell therapy centers in the federal state of Saxony,
- reside within the regular service area (≤ 200 km) of one of the three centers,
- own a smartphone and are able to use it,
- have provided written informed consent to participate in the SPIZ RCT, including all components of the intervention, and.
- are covered by the German statutory health insurance system.

##### Patients meeting the following **exclusion criteria** are not included to the SPIZ study

- more than eight days have elapsed since hospital discharge following alloHCT or CAR T-cell therapy,
- insufficient German language proficiency to use the SMILe App and to complete study questionnaires (e.g., QoL or program satisfaction),
- medical conditions that render participation in an intensified outpatient post-treatment care pathway infeasible.

The secondary study population consists of patient advocates, and healthcare providers including hematologists, case managers and onco nurses at the three participating cell therapy centers as well as community-based hematologists, being involved in the SPIZ program.

### Technical implementation of digital elements

The SMILe App enables patients after alloHCT to systematically document their health status continuously and in real-time, including vital parameters and disease or cellular therapy associated symptoms. SMILe Care provides an interface enabling onco nurses to review these patient-reported outcomes [15, 16]. For the SPIZ program, the app was augmented with additional functionalities to address the specific medical needs of patients following CAR T-cell therapy. In addition, the integrated patient education lexicon was substantially expanded to include more comprehensive information regarding alloHCT and CAR T-cell therapy. Additionally, a secure text-based communication function enabling patients to contact the treating center was implemented. The app itself does not generate automated alerts or apply predefined alert thresholds or algorithms. Data entered into the app are generally reviewed by onco nurses on the morning of the next working day. Individual abnormal measurements and clinically relevant trends are assessed in accordance with the participating center’s standard operating procedures. If an abnormality is identified, the responsible physician at the respective center is contacted immediately to determine further management. If clinically indicated, the patient is subsequently contacted without delay and directed to an appropriate level of care, e.g. local emergency medical services, the patient’s outpatient hematologist or primary care physician, cellular therapy center hematologist or the emergency department.

The app is not intended for emergency communication and is not monitored continuously or outside the specified review periods. During onboarding, participants are explicitly instructed not to use the app for urgent concerns. During case management service hours (8 a.m. to 3 p.m.), urgent concerns are immediately escalated to the responsible physician. Outside these hours, participants are instructed to follow the established pathways of their treating center, including contacting its on-call service or, depending on the severity of the concern, contacting local emergency medical services or presenting to an emergency department. These instructions are consistent with the center-specific standard operating procedures and routine discharge information provided following cellular therapy.

Video consultations are conducted via institutional, secure virtual consultation platforms, e.g. CARY medical by Webex. Online interdisciplinary case conferences are held every two weeks and documented via a virtual tumor board platform.

### Enrollment, randomization and patient pathway

**Fig. 2:**
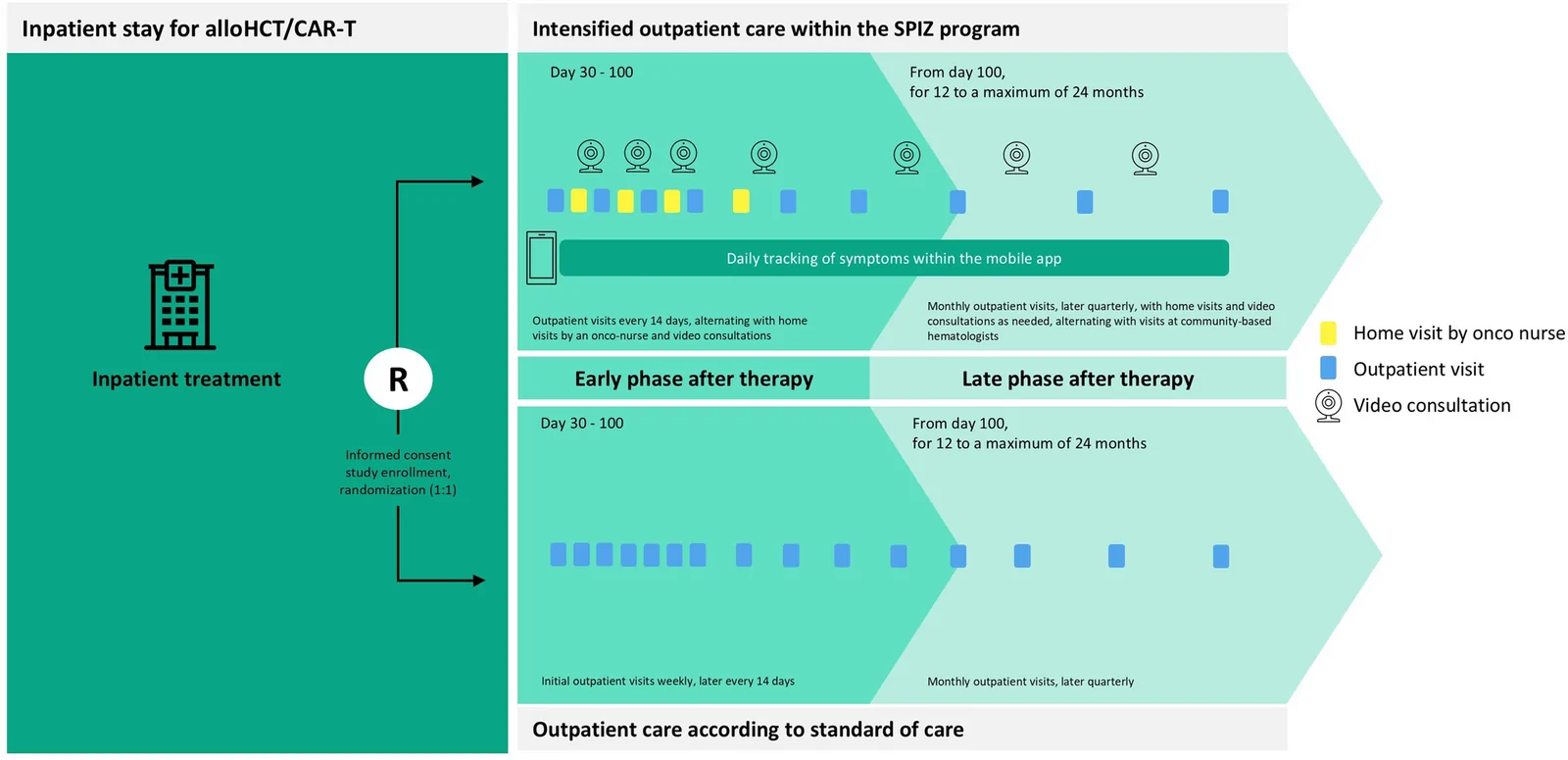
Patient pathway in the SPIZ RCT: After enrollment, patients are randomized in a 1:1 ratio to either the SPIZ group or the control group. In the SPIZ group, home visits by onco nurses are carried out as needed and alternate with routine outpatient clinic visits. Patients document vital signs and symptoms daily via the SMILe App, which are monitored by onco nurses. Regular video consultations with hematologists at the cell therapy center take place, either following home visits or on demand. Patients in the control group receive routine post-treatment care. Onco nurse: oncology nurse; R: randomization

After enrollment, which takes place not later than eight days following discharge from the primary inpatient stay for cellular therapy, patients are randomly assigned in a 1:1 ratio to either the SPIZ program or the control group. The randomization is stratified according to patient age (< 60 years or ≥ 60 years) for each of the three cellular therapy centers individually using the REDCap (Research Electronic Data Capture)-based project database. Prior to the commencement of the study, age-group-specific randomization lists have been created through block randomization, employing permuted blocks of variable length. These randomization lists are prepared by the Dresden Coordination Center for Clinical Trials, which is also responsible for the preparation and administration of the REDCap project database. The randomization result is displayed to study staff in REDCap once informed consent and eligibility based on the inclusion and exclusion criteria have been documented. Depending on group allocation, patients proceed through either the SPIZ pathway or standard routine care (Fig. 2). Patients are scheduled to receive treatment for a minimum duration of 12 months and a maximum duration of 24 months.

Home visits by an onco nurse are scheduled at predefined time points (supplementary Figs. 1 and 2) and constitute a planned component of the intervention. Together with a subsequent video consultation, each scheduled home visit replaces a routine outpatient visit at the center. Additional home visits may be arranged when clinically indicated. Multidisciplinary case conferences are scheduled at week 6, week 12, month 6, month 12 and month 24 (supplementary Figs. 1 and 2).

Home visits by an onco nurse combined with video consultations replace some visits to the cell therapy center in comparison with standard of care. Besides that, participants in the intervention group continue to receive all routinely indicated outpatient visits, diagnostic assessments, treatments, and access to emergency care at their treating center. Additional home visits, video consultations, app-supported monitoring, case management, and multidisciplinary case conferences are supplementary components. Participants in the control group receive usual standard of care without the protocol-specific SPIZ components.

Missed app entries, unsuccessful attempts to contact a participant, and the outcome of each contact attempt are documented in the hospital information system and, separately, in the REDCap database. When app entries are missing, the case manager or onco nurse make repeated attempts to contact the participant. If the participant remains unreachable, the responsible physician is informed, and further action is taken in accordance with the respective center’s standard operating procedures.

### Hypotheses and outcomes

The primary hypothesis is that patients receiving the SPIZ intervention following cellular therapy (intervention group) experience better health outcomes in terms of lower mortality and fewer hospital readmissions than patients receiving routine clinical care (control group). The target population comprises all adult patients with hematologic diseases who undergo alloHCT or CAR T-cell therapy at one of the three participating centers.

Efficacy will be evaluated using a composite primary endpoint comprising mortality and disease-related rehospitalization. The latter is defined as unplanned rehospitalization due to complications typically associated with either the underlying hematologic disease (e.g., disease progression) or cellular therapy. Such complications include infections and organ toxicities following either therapy, GvHD following alloHCT, and CRS or neurotoxicity, such ICANS, following CAR T-cell therapy. The primary endpoint will be evaluated by confirmatory statistical analysis 12 months after cellular therapy.

Secondary hypotheses with corresponding outcomes and data sources are listed and described in Table 1.

**Table 1:** Overview of the secondary hypotheses. The four secondary hypotheses to be examined are listed along with their corresponding secondary outcomes and the respective data sources or scales

Secondary hypotheses	Secondary outcomes	Data source / scale
a) SPIZ leads to improved longitudinal clinical outcomes	◼ Mortality ◼ Complications, e.g., infections, GvHD (alloHCT), CRS (CAR T-cell therapy), neurotoxicity (CAR T-cell therapy), relapse, and death assessed by incidence and duration (as feasible) ◼ Rates and duration of unplanned rehospitalizations ◼ Symptom burden, including nutrition, body weight, body temperature, skin changes, bowel habits, and neurological symptoms ◼ Laboratory values ◼ Therapy adjustments ◼ Conducted assessments, e.g. staging, pulmonary function testing, echocardiography	REDCap-based project database, SMILe system
b) SPIZ leads to improvements in QoL and patient empowerment	QoL assessed using established questionnaires	EORTC QLG Core Questionnaire QLQ-C30 and module for low-grade non-Hodgkin lymphoma QLQ-NHL-LG20 [17], NCCN Distress-Thermometer (German version) [18]
	Empowerment: ◼ Shared decision making ◼ Pathway adherence ◼ Self-assessment of health status ◼ Patients’ level of information about the course of treatment and possible treatment options	Questionnaire survey using self-developed items; Qualitative interviews with patients
c) SPIZ increases satisfaction among both patients and healthcare providers with a high level of acceptance	Patients’ perspective ◼ Satisfaction with the program and/or specific components ◼ Causes for satisfaction/dissatisfaction ◼ Suggestions for improvement of the SPIZ program	Questionnaire survey with modified PSCC (Patient Satisfaction with Cancer Care*)* [19] and self-developed items; Qualitative interviews with patients
	Healthcare providers’ perspective ◼ Satisfaction with the program and/or specific components ◼ Satisfaction with communication between different healthcare provider groups ◼ Causes of satisfaction/dissatisfaction	Qualitative interviews with healthcare providers
d) SPIZ is cost-effective from the perspective of the statutory health insurance system	◼ Rates and costs of rehospitalizations ◼ Costs of outpatient treatments and medication ◼ Travelling costs ◼ Level of care related and palliative care costs	REDCap-based project database, Statutory health insurance routine data from AOK PLUS

*alloHCT* allogeneic hematopoietic cell transplantation, *CAR* T-cell therapy, chimeric antigen receptor T-cell therapy, *GvHD* graft-versus-host disease, *QoL* Quality of Life, *REDCap* Research Electronic Data Capture, *SMILe* SteM-cell-transplantatIon faciLitated by eHealth, *SPIZ* cross-sector care for patients with hematological diseases following innovative cell therapy

### Sample size

Sample size calculation for the primary study population is based on the primary endpoint. The literature available at the time of sample size planning showed that there was a rehospitalisation rate of 55% after alloHCT in a German population [10]. Given that deceased patients were typically hospitalised beforehand, it can be inferred that the 55% figure accurately represents the routine clinical care. The SPIZ program should reduce the mortality or disease-specific rehospitalisation rate to 35% or less. The case number calculation was performed with a one-sided Fisher test. Given a significance level of 5% and a yielded power of 80%, it is assumed that 143 patients in the intervention group and 143 patients in the control group are required (calculated with GPower 3.1). With an estimated drop-out rate of 5%, 302 patients must be included.

While disease- and cell therapy-specific rehospitalization or death includes all complications typically associated with the underlying hematological condition or cell therapy, other reasons for rehospitalization or death are defined as independent, e.g., joint replacement due to arthrosis, or an accident. The analysis of the primary endpoint will be conducted one year after primary hospital discharge of the last enrolled patient. Death or rehospitalization are considered equivalent primary endpoints.

Recruitment of health care providers involved in SPIZ (secondary study population) for twelve qualitative interviews starts 18 months after initiation of patient enrollment.

### Data collection and management

Primary and secondary outcomes are collected with six prospective primary data sources and one secondary data source.

Data source 1 [DS1] is the project database based on REDcap to document patient cases, events and process data, e.g., sociodemographic data, outpatient appointments, and clinical outcomes at baseline and 6, 12, 18 and 24 months after cellular therapy. Baseline clinical status is defined as the patient’s clinical status on the day of discharge from the index hospitalization. Data quality is monitored through automated verification of the recorded data, notifications regarding missing entries, and regular monitoring at the three study centers.

Data source 2 [DS2] are validated QoL surveys. To evaluate the QoL of patients after alloHCT or CAR T-cell therapy, the survey instruments EORTC QLG Core Questionnaire (EORTC QLQ-30) and the module for low-grade non-Hodgkin lymphoma (EORTC QLQ-NHL-LG20) [17] as well as the German version of the NCCN Distress Thermometer [18] are used. QoL data is evaluated at baseline and 6, 12, 18 and 24 months after discharge (Table 1).

Data source 3 [DS3] is an online-survey to evaluate satisfaction of patients with the SPIZ program. We use an adjusted version of the validated survey instrument Patient Satisfaction with Cancer Care (PSCC) [19] to evaluate satisfaction with the follow-up care in general for both intervention and control group. Additionally, we developed specific questions for each SPIZ component to measure satisfaction within the intervention group. These questionnaires are sent to patients via e-mail as an online survey 12 months after discharge and at the end of the study.

Data source 4 [DS4] is the SMILe App. Patients in the intervention group are provided with the SMILe App for their mobile phones and continuously document their health status, including vital parameters and symptoms (e.g., body temperature, body weight), and well-being. With the SMILe App, data is stored over time.

With data source 5 [DS5] and data source 6 [DS6] qualitative empirical data is collected. [DS5] comprises semi-structured individual telephone interviews with patients and health care providers. We conduct twenty-four qualitative interviews with patients from the three participating cell therapy centers (four interviewees from the intervention group and four interviewees from the control group from each center, respectively) about their experiences and satisfaction with their post-treatment care approximately six months after discharge. In order to reflect the heterogeneity of the patient group, the selection occurs based on purposeful sampling i.e. with respect to age, gender, type of therapy, distance between place of residence and hospital, level of education and occupational status. In addition, we conduct twelve interviews with healthcare providers across all three participating study sites. That is to say, at each site, one hematologist from the cell therapy center, one community-based hematologist, one onco nurse and one case manager will be interviewed to integrate the perspectives of all in post-treatment care involved healthcare providers. All interviews will be audio recorded and transcribed verbatim with the help of the speech recognition software AlphaSpeech (Version 1.9.0, hosted on servers of TUD Dresden University of Technology), followed by manual corrections. A focus group interview with healthcare providers of the three cell therapy centers will be [DS6]. The focus group interview will be audio recorded and transcribed as described for the qualitative interviews above.

Routine data from the participating statutory health insurance provider, AOK PLUS, will be used as data source 7 for all patients insured with this provider [DS7.

Fidelity measures include successful app onboarding, the frequency of app entries and data reviews, the number and type of case management contacts, home visits and video consultations, participation in case conferences, completion of scheduled assessments, and deviations from the planned intervention pathway. As the intensity of several components is tailored to participants’ clinical needs, no minimum number of home visits, video consultations, or case conferences is required. Intervention delivery is considered to have commenced once the participant has completed SPIZ onboarding, is discharged from index hospitalization, and has used at least one component of the SPIZ intervention. To minimize contamination, the SPIZ-specific components are organized separately and provided only to participants allocated to the intervention group. Control-group participants are not enrolled into the SMILe App, assigned to a SPIZ case manager, proactively monitored by the onco nurses, or included in protocol-specific home visits, video consultations, or case conferences. Clinically necessary care will never be withheld from either group. All potential primary endpoint events will be adjudicated by two board-certified hematologists who are blinded to treatment allocation. Any disagreements will be resolved by consensus or, if necessary, by a third board-certified hematologist who is also blinded to treatment allocation.

### Data analysis regarding primary hypothesis and secondary hypothesis a)

The confirmatory test of the primary outcome at 12 months will be performed using Fisher’s exact test. All outcomes of secondary hypothesis a) are presented descriptively for the intervention and control groups according to the respective survey dates. The same applies to the primary combined outcome at time points 6, 18, and 24 months. Metric values will be compared between groups in terms of their means or, in the case of skewed distributions, their medians; categorical values will be compared in terms of their distributions. For events (death/ complication/ hospitalisation), survival time analyses and hazard ratios will be calculated. Logistic or Poisson regressions will be used to estimate effect size measures (odds ratios or relative risks). In addition, the control and intervention groups will be compared with regard to potential confounders. Should serious biases occur despite randomization, these will be addressed as appropriate through adjustments, subgroup analyses, or sensitivity analyses.

### Data analysis of secondary hypothesis b) and c)

We descriptively analyze quantitative survey data for assessing QoL [DS2] and satisfaction of patients with the SPIZ program [DS3] as described for the testing of secondary hypothesis a). For the intervention group, a comparison will also be made with the services provided for in the pathway.

To further evaluate patients’ satisfaction/dissatisfaction with SPIZ and its components, the transcripts of the 24 qualitative interviews with patients [DS5] will be qualitatively analysed using deductive and inductive coding [20] and analysed computer-assisted with MAXQDA 24 (VERBI Software 2023). In order to achieve intersubjective comprehensibility [21] two researchers conduct the coding process consecutively and independently. Any differing or unclear coding will subsequently be discussed and resolved jointly.

Satisfaction of health care providers with the SPIZ program will be assessed with a qualitative content analysis of the interview transcripts as described above. Based on the results of the qualitative interview analysis, we explore and discuss potential facilitators and barriers to the implementation of the SPIZ program into routine healthcare in a focus group with health care providers [DS6]. The focus group will be analysed as described above for qualitative individual interviews.

### Data analysis of secondary hypothesis d)

The health economic evaluation of the SPIZ program focuses on a comparison between the intervention and control groups in terms of inpatient stays and the resulting costs for the statutory health insurance AOK PLUS. The latter is the statutory health insurance fund with the most insured persons in the catchment area, thus ensuring a sufficient number of test subjects for analysis.

These are standardized over the entire observation period in terms of number of stays, average length of inpatient stays, and costs per observation year.

Further analyses relate to the comparison between the two groups in terms of selected outpatient costs, costs for medications dispensed in pharmacies, patient travel costs, care level, and number of patients in palliative care.

### Principles of the analyses

All analyses within the scope of the SPIZ evaluation are carried out in the intention-to-treat population. Missing values are identified, and their underlying mechanisms are examined in terms of randomness. When values are missing, the individuals in question are excluded from the respective sub-analyses. The results of the evaluation are published in full, transparently, and independently of the outcome of the evaluation. The method chapter is based on the SPIRIT reporting guidelines [22]. Reporting is based on the CONSORT and TIDieR guidelines [23, 24].

## Results

The study was approved by the institutional review board of the Medical Faculty of TUD Dresden University of Technology (BO-EK-408092023), the institutional review board of the Medical Faculty of Leipzig University (405/23-lk X), and the Chamber of Physicians of Saxony (EK-BR-130/23 − 1). The study has been registered in the German Clinical Trials Register (DRKS-ID: DRKS00034454). Written informed consent was obtained from all participants. The study is conducted in accordance with the World Medical Association Declaration of Helsinki. A study-specific data protection concept has been developed and implemented. Any protocol amendments will be registered in the German Clinical Trials Register.

Following the establishment of the required study personnel and infrastructure at the three cell therapy centers, the first patient was enrolled on January 16, 2024. After a recruitment period of two years, enrollment was completed on January 29, 2026. To allow sufficient time for patient training regarding the SMILe App and usage of video consultations in the intervention group, patients were usually enrolled and randomized several days prior to the anticipated discharge. Therefore, usually some time elapsed until study commencement, which was scheduled at the first outpatient visit. Upon reaching the planned sample size of 302 patients, we observed that only 149 intervention group patients reached study commencement, compared to 153 patients in the control group. Recruitment was therefore continued according to the randomization lists, finally leading to 153 patients in each group, respectively. Given the minimum post-treatment follow-up period of 12 months, the first study results are expected in mid-2027.

## Discussion

The SPIZ study will evaluate a structured model of post-treatment care after alloHCT and CAR T-cell therapy in comparison with routine care in a multicenter RCT, thereby unlocking the potential of intensified support by specialized healthcare providers combined with digital components. Through telemonitoring, we aim to facilitate the early detection of complications and enable timely, targeted interventions and increase the QoL of the patient by reducing the timely intensive visits in the center. By providing additional personnel resources and conducting home visits, we aim to relieve the burden on patients who are often severely affected by their underlying disease and cellular therapy, without bypassing medically necessary visits to the cell therapy center. This approach has the potential to reduce travel burden for many patients, especially those living in rural areas, as it is true for cellular therapy patients in Saxony, with the potential of transferring it to other less densely populated states and countries. The option of short-notice video consultations will allow for rapid scheduling of minor follow-up appointments.

A potential limitation of the study is that the digital components of the intervention may pose a barrier for older patients. However, alloHCT is typically offered to patients younger than 75 years. In our experience, the majority of these patients own a smartphone and are able to use it. Limited digital literacy, insufficient access to suitable devices or a stable internet connection, socioeconomic disadvantage, limited German-language proficiency, and visual, hearing, cognitive, or motor impairments may reduce patients’ ability or willingness to participate. Moreover, some patients may depend on caregiver assistance to use the digital components of the intervention. The present study does not systematically assess the effects of these factors on recruitment, adherence, or outcomes. Consequently, patients facing digital, socioeconomic, linguistic, or disability-related barriers may be underrepresented. Due to requirements related to the health-economic analysis, patients with private health insurance are excluded from participation. According to the 2024 private insurance reporting in 2024, privately insured individuals accounted for 6.2% of the population in Saxony [25]. Thus, the numerical effect of this exclusion on representativeness is likely limited, although systematic differences between insurance groups cannot be ruled out. Importantly, private health insurance would not constitute an exclusion criterion if the intervention will be implemented into routine clinical care.

Central coordination by case managers is intended to ensure seamless post-treatment care for all patients, irrespective of their individual resources. In addition, support from onco nurses in the patients’ home environment may facilitate the early identification of potential safety risks and enable better involvement of the patients’ family. Regular virtual case conferences are expected to strengthen collaboration with community-based hematologists, thereby supporting cross-sectoral continuity of the care model. The needs-based integration of psycho-oncology and social counseling services may complement medical care and aims to provide comprehensive support to intensively treated patients across all aspects of life.

The approach of a structured care model may facilitate comparability and standardization with post-treatment care after cellular therapy. It also incorporates current developments in telemedicine and digitalization, which are being progressively advanced in Germany, for example through the telematics infrastructure aimed at improving connectivity among healthcare providers [26]. In addition, the digitalization of hospitals is being driven forward, particularly through the Hospital Future Act (Krankenhauszukunftsgesetz) [27].

To capture these aspects in the evaluation, the study design incorporates the following considerations: The selection of interview participants for the qualitative interviews was conducted based on a sampling strategy that incorporated not only sociodemographic criteria (e.g., age, gender) and the type of therapy (alloHCT or CAR T-cell therapy) but also factors such as the distance between the patient’s residence and the cell therapy center, educational background, marital status, and employment status. This approach ensures that the diverse life-world contexts of the patients are taken into account in the evaluation of satisfaction with the SPIZ program. It also may give insights whether patients in different circumstances benefit differently from the SPIZ program and its components. Furthermore, with regard to varying educational levels, the study examines the extent to which the information provided and the structure of the program are comprehensible to all patients.

The use of a mixed-methods design to evaluate patient satisfaction with the SPIZ program enables the collection of both individual patient experiences with the specific components of the SPIZ program and overall patient satisfaction with the intervention as a whole. The qualitative-empirical interviews with service providers allow for a deeper understanding of their experiences with the new form of care. In particular, the direct comparison of the current standard of post treatment care following cellular therapy with the SPIZ program can reveal areas where SPIZ contributes to improvement post-treatment care in the future. Through interviews with hematologists at the cell therapy center and in community-based practice, we aim to assess the extent to which SPIZ enhances cross-sectoral collaboration and to identify the areas in which this occurs.

Overall, the present study addresses the substantial need for intensive and structured post-treatment care following cellular therapy, particularly in rural regions, by implementing an interdisciplinary, digitally supported, cross-regional and cross-sectoral care model. Owing to the specific funding structure, a positive evaluation would enable long-term reimbursement of the SPIZ program for all patients insured under the statutory health insurance system in Germany, thereby improving care sustainably for this vulnerable patient population.

## Supplementary information

Below is the link to the electronic supplementary material.

Supplementary figure 1: Post-treatment care plan following allogeneic hematopoietic cell transplantation (alloHCT). Abbreviations: acc: according; alloHCT: allogeneic hematopoietic cell transplantation; ALT: alanine aminotransferase; AP: alkaline phosphatase; AST: aspartate aminotransferase; CBH: community-based hematologist; CMV: cytomegalovirus; CRP: C-reactive protein; DXA: dual-energy X-ray absorptiometry; ECOG: Eastern Cooperative Oncology Group; eGFR: estimated glomerular filtration rate; GGT: gamma-glutamyl transferase; GvHD: graft-versus-host disease; m: month(s); IgA/IgG: immunoglobulin A/G; NIH: National Institutes of Health; QoL: quality of life; PCR: polymerase chain reaction; SARS-CoV-2: severe acute respiratory syndrome coronavirus 2; SOP: standard operating procedure; w: week(s)

Supplementary figure 2: Post-treatment care plan following chimeric antigen receptor T (CAR T)‑cell therapy (CAR‑T). Abbreviations: Acc.: according; ALT: alanine aminotransferase; AP: alkaline phosphatase; AST: aspartate aminotransferase; CAR-T: chimeric antigen receptor T-cell therapy; CBH: community-based hematologist; CMV: cytomegalovirus; CRP: C-reactive protein; DXA: dual-energy X-ray absorptiometry; EBV: Epstein-Barr virus; ECOG: Eastern Cooperative Oncology Group; eGFR: estimated glomerular filtration rate; GGT: gamma-glutamyl transferase; GvHD: graft-versus-host disease; m: month(s); IgA/IgG: immunoglobulin A/G; NIH: National Institutes of Health; QoL: quality of life; PCR: polymerase chain reaction; SOP: standard operating procedure; w: week(s)

## Data Availability

The trial protocol and statistical analysis plan are available from the authors upon reasonable request. The final data may be requested from the authors upon completion of the study; however, participant data is subject to specific data protection requirements.

## Declarations

### Trial registration

German Clinical Trials Register (DRKS-ID: DRKS00034454) on January, 31, 2025 (https://drks.de/search/en/trial/DRKS00034454).

## Abbreviations

alloHCT: allogeneic hematopoietic cell transplantation
CAR T-cell therapy: chimeric antigen receptor T-cell therapy
e.g.: for example (exempli gratia)
EORTC: European Organization for Research and Treatment of Cancer
G-BA: Federal Joint Committee (Gemeinsamer Bundesausschuss)
GvHD: graft-versus-host disease
i.e.: that is (id est)
NRM: non-relapse mortality
Onco nurses: oncology nurses
PSCC: Patient Satisfaction with Cancer Care
QoL: quality of life
RCT: randomized clinical trial
REDCap: Research Electronic Data Capture
SMILe: SteM-cell-transplantatIon faciLitated by eHealth
SPIZ: cross-sector care for patients with hematological diseases following innovative cell therapy (Sektorenübergreifende Versorgung von Patientinnen und Patienten mit hämatologischen Erkrankungen nach innovativer Zelltherapie)
